# Use of Clinical EMR Systems for Targeted Recruitment of Older Adults for Research: Facilitators and Barriers

**DOI:** 10.1093/geroni/igaf122.4342

**Published:** 2025-12-31

**Authors:** Lisa Kenyon-Pesce, Roshanak Sharafieh, Karina Berg, Tina Ferrarotti, George Kuchel, Richard Fortinsky, Iman Al-Naggar, Julie Robison

**Affiliations:** UConn Health, Farmington, Connecticut, United States; UConn Health, Farmington, Connecticut, United States; UConn Health, Farmington, Connecticut, United States; UConn Health, Farmington, Connecticut, United States; UCONN HEALTH, Farmington, Connecticut, United States; University of Connecticut, Farmington, Connecticut, United States; UConn Health, Farmington, Connecticut, United States; University of Connecticut, Farmington, Connecticut, United States

## Abstract

Identifying participants with specific diagnoses through the Electronic Medical Record (EMR) represents one of the most promising strategies for targeted research recruitment. Given the remarkable heterogeneity of the aging population in terms of functional and cognitive status, clusters of co-morbidities, medication use, decline of physical function and increased risk of frailty, EMR-based approaches should be especially helpful for recruitment in observational and interventional studies involving older adults. However, the implementation of this approach faces real-world obstacles that prevent expensive institutional investments in EMRs from meeting their full potential. These barriers include the absence of functional and accurate data in EMRs, provider burden and burnout resulting from EMR alert fatigue, compliance and regulatory issues, as well as occasional misperceptions and pushback among institutional leaders. This presentation aims to discuss facilitators and barriers of utilizing EMR alerts as a recruitment method for identifying patients for research studies. A case study highlights the challenges and benefits of using Electronic Medical Records (EMR) systems to recruit older adults with early-stage lower extremity wounds. In addition, we discuss potential solutions, such as better stratifying frailty, function, and care preferences, while overcoming regulatory obstacles in the effective implementation of these important tools.

